# Association of parental education with offspring psychiatric diagnoses, violent crimes, and suicidal behavior: a nationwide Swedish quasi-experimental study

**DOI:** 10.1186/s12916-026-04719-w

**Published:** 2026-02-26

**Authors:** Mengping Zhou, Henrik Larsson, Brian M. D’Onofrio, Mikael Landén, Paul Lichtenstein, Erik Pettersson

**Affiliations:** 1 Department of Medical Epidemiology and Biostatistics, Karolinska Institutet, Box 281, Stockholm, 171 77 Sweden; 2 School of Medical Sciences, Örebro University, Örebro, Sweden; 3 Department of Psychological and Brain Sciences, Indiana University, Bloomington, IN USA; 4 Institute of Neuroscience and Physiology, The Sahlgrenska Academy at Gothenburg University, Gothenburg, Sweden

**Keywords:** Swedish education reform, Parental education, Mental health problems, Quasi-experimental study, Fuzzy regression discontinuity design

## Abstract

**Background:**

Higher parental educational attainment is associated with a reduced risk of mental health problems in offspring. However, these associations might partially reflect unmeasured confounding rather than causal effects.

**Aims:**

To investigate the association between parental education and offspring psychiatric diagnoses, violent crimes, and suicidal behavior, after isolating unmeasured confounding.

**Methods:**

We analyzed data from 1,047,275 offspring to 701,350 parents born between 1945 and 1955 in 926 Swedish municipalities. The exposure was parental years of schooling. The outcomes were 14 psychiatric diagnoses, court convictions of violent crimes, and suicidal behavior in the offspring. We first estimated the association between parental years of schooling and offspring outcomes using stratified Cox regression. Subsequently, leveraging a Swedish education reform that extended compulsory schooling from 7 to 9 years as a quasi-experiment, we compared parents educated immediately before and after the reform's implementation. This approach allowed us to estimate the reform's impact on offspring outcomes while controlling for potential unmeasured confounders. To estimate the causal effect of the parental education on offspring outcomes, we applied a fuzzy regression discontinuity (RD) design, that is, we used the Swedish education reform as an instrument for parental years of schooling. If the RD model assumptions are met, this design identifies the causal effect of education among those who planned to quit school after completing the minimum compulsory requirement.

**Results:**

Parental years of schooling were negatively associated with most of the 14 psychiatric diagnoses, violent crime, and suicidal behavior in their offspring. However, when comparing children of parents exposed to the reform versus those not exposed, there were no significant differences in these outcomes (e.g., any psychiatric diagnosis: hazard ratio = 1.00, 95% CI 0.99–1.02). Consistently, RD estimates of the effect of parental years of schooling on offspring outcomes were close to the null and not statistically significant after correcting for multiple testing.

**Conclusions:**

Among parents who planned to quit school after completing the minimum compulsory requirement, the negative association between longer years of parental schooling and offspring mental health problems and behavioral outcomes likely reflected unmeasured confounding.

**Supplementary Information:**

The online version contains supplementary material available at 10.1186/s12916-026-04719-w.

## Background

Higher parental educational attainment is negatively associated with psychiatric disorders and suicidal behaviors in offspring [1–6]. Although researchers have suggested potential mediators including increased parental income, delayed age at first birth, better parental health, enhanced parenting skills, and/or improved education in the children [7–11], family and twin studies show that at least some of the association might also reflect confounding by unmeasured shared genetic and environmental factors. For example, a longitudinal study (*N* = 4693) found that the association between maternal education and offspring attention deficit hyperactivity disorder (ADHD) attenuated after controlling for polygenic scores and disappeared after adjusting for twin-based heritability [12]. Similarly, a large children-of-twins/siblings study (*N*_offspring_ = 34,958 children, *N*_parents_ = 28,372 twins/siblings), which controlled for unmeasured familial confounding shared by cousins, observed that parental educational attainment was not associated with offspring depressive symptoms (though it remained associated with offspring ADHD symptoms) [13].

While genetically informed designs can control for shared unmeasured familial confounding, residual genetic confounding might remain when comparing cousins (at least when the respective parents are dizygotic twins or regular siblings). Furthermore, within-family comparisons remain biased by unmeasured confounds that are not shared by family members. In contrast, by exploiting exogenous variation in exposures, instrumental variable (IV) designs control for all unmeasured confounding (albeit at the cost of sometimes making strong assumptions, depending on the instrument) [14, 15]. Studies using instruments such as parental long-standing illness [16] and education-related genetic variants [17] have largely reported null effects of parental education on offspring mental health outcomes during childhood or adolescence. However, a study that used local educational infrastructure variables (i.e., local tuition fees, distance to college, and local labor market) as instruments found a protective effect of increased maternal education on offspring behavioral problems [18].

Thus, studies using both within-family comparisons and IV designs have reached somewhat inconsistent findings, potentially because of residual confounding or violated model assumptions. In contrast, studies drawing on education reforms primarily assume that individuals before and after the implementation are similar, potentially making them better suited to examine associations free from unmeasured confounding. In two education reform studies in Denmark (which increased compulsory schooling from 7 to 9 years) and the UK (which raised the minimum school leaving age from 14 to 15), respectively, increased compulsory schooling had a positive effect on parental income, but there were no effects on their children’s physical or mental health outcomes [7, 19].

While these studies comprehensively measured physical health, their assessment of mental health was limited in four ways. First, the mental health assessments were limited to service utilization or parent-reported overall conditions without specifying types, respectively, potentially missing heterogeneous effects [20]. Second, both studies focused on mental health outcomes during childhood and adolescence. Third, neither considered behavioral outcomes such as suicide or criminality. Lastly, the Danish study focused exclusively on maternal education due to the weak reform effects on paternal education, leaving the causal impact of paternal education on child health unexplored. Addressing one of these limitations, that is, the relatively short follow-up period, a more recent education reform study in Germany (which increased compulsory schooling from 8 to 9 years) observed that one additional year of maternal schooling was associated with daughter-reported poorer mental health by adulthood (though there were no effects for sons) [21].

The goal of this study was to remedy past limitations by investigating the association of maternal and paternal education, as instrumented by a Swedish education reform that increased the minimum schooling from 7 to 9 years, with a wide array of offspring mental health problems and behavioral outcomes—including clinically recorded psychiatric disorders, court convictions of violent crimes, and suicidal behavior—by middle adulthood.

## Methods

### Swedish compulsory schooling reform

As described by Holmlund [22, 23], the Swedish education reform extended compulsory schooling from 7 to 9 years to increase equality of opportunity and meet growing educational demands. The reform was gradually implemented across Swedish municipalities between 1949 and 1962, which constitutes a quasi-experiment to evaluate its effect. As Swedish children traditionally begin first grade the year they turn 7, the reform encompassed birth cohorts from 1938 (the first cohort affected, entering fifth grade in 1949) to 1955 (the last cohort affected, beginning first grade in 1962).

### Study population

We first identified all individuals born in Sweden between 1945 and 1955 from the Total Population Register. We excluded (1) municipalities where the reform cohort year (i.e., the first cohort affected by the reform in a given municipality) occurred outside 1938–1955; (2) municipalities where the reform cohort year occurred before or in 1945, as all individuals in such municipalities were exposed to the reform and did not contribute to the analysis; (3) three major cities (Stockholm, Gothenburg, and Malmö) and six municipalities (Hälsingborg, Jönköping, Linköping, Skellefteå, Sundbyberg and Södertälje) where the reform was introduced gradually; and (4) individuals who died before the age of 18 or had missing data on home municipality or educational attainment. This left 71% of the total sample.

We then linked these individuals to their offspring born between 1970 and 2000 (using the Multi-Generation Register) and to several other national registers to obtain their psychiatric diagnoses, violent crimes, and suicidal behavior. Table S1 in Additional file 1 presents a detailed description of these registers. In the offspring generation, we excluded stillbirths, individuals with congenital malformations, and those who died neonatally. We followed the offspring from birth to the occurrence of the outcome, emigration from Sweden, death, or December 31, 2013, whichever came first.

The Swedish Ethical Review Authority approved this study, and informed consent was not required because this was a register-based epidemiologic study.

### Exposure

The exposure was parental years of schooling, which represented the average number of years of educational attainment [24]: 7 years for compulsory education less than 9 years (i.e., old compulsory school, *folkskola*), 9 years for compulsory education of 9 years (i.e., new compulsory school, *grundskola*), 11 years for short high school (i.e., upper secondary schooling), 12 years for long high school, 14 years for short university, 15.5 years for long university, and 19 years for postgraduate. Information on educational attainment level was derived from the 1970 and 1990 Population and Housing censuses.

### Outcomes

For the outcomes, we extracted 14 types of psychiatric diagnoses, including schizophrenia, bipolar disorder, alcohol-related disorders, drug-related disorders, depression, anxiety, obsessive–compulsive disorder (OCD), autism spectrum disorder (ASD), posttraumatic stress disorder (PTSD), tic disorder, oppositional defiant disorder (ODD), ADHD, learning disorders, and intellectual disability. To compare with previous studies [7, 19], we additionally created an outcome that included any psychiatric diagnosis (ICD-8, 290–319; ICD-9, 290–319; ICD-10, F00-99). We also included court convictions of violent crimes and suicidal behavior (including suicide attempts and death by suicide). Table S2 in Additional file 1 presents the ICD code, a description of convictions classified as violent crimes, and the minimum cutoff age for each outcome (prior to which a diagnosis might be considered unreliable). Table S1 in Additional file 1 presents the corresponding registries from which these outcomes were extracted.

### Assign the reform status

We assigned the reform status using the method outlined by Holmlund [22]. Briefly, this involves identifying the cohort year in which the reform was implemented at a municipality level (i.e., the reform cohort year) using the Swedish population register data. To accomplish this, we first dropped all individuals with education levels higher than the new compulsory minimum, which left us with observations of only the old compulsory minimum (assigned as 0) and the new compulsory minimum (assigned as 1). Second, we collapsed this data by birth cohort and municipality to identify the reform cohort year. Within each municipality, we identified the year in which the reform was implemented (i.e., the reform cohort year) according to when there was a discontinuous jump from below to above 0.5 (with no subsequent drop back below 0.5). We then determined individuals’ reform status based on their birth year relative to the reform cohort year. Those born before the reform cohort year were identified as unexposed (coded as 0), and those born during or after the reform cohort year were identified as exposed (coded as 1). Information on municipality was derived from the 1960 Population and Housing census.

As outlined by Holmlund, this approach is subject to potential measurement errors [22]. First, this assignment assumes that individuals are in the right grade according to their age, which we subsequently addressed in a sensitivity analysis (see below). Second, individuals might change their residences between birth and school age, with residential mobility estimated at approximately 13% [25]. Third, some individuals might have the birth hospital municipality recorded as their place of birth rather than their actual place of residence, with a misclassification rate around 12% ^26^. Nevertheless, a study using the Individual Statistics database from the Institute of Education at Gothenburg University observed 93% accuracy in this reform assignment approach, suggesting that these potential misclassification errors likely have limited impact on the overall results [26].

### Statistical analyses

First, we estimated the association between parental years of schooling and offspring outcomes using the stratified Cox regression to estimate the hazard ratio (HR), with age as the underlying time scale and each municipality as a separate stratum. We adjusted for parental birth year, sex, and offspring birth year by adding dummy variables. We estimated robust standard errors (SEs) by clustering on the family level.

We then estimated the association between the reform and the offspring outcomes (typically labeled reduced form, or an intent-to-treat [ITT] effect) using the stratified Cox regression model described above. The corresponding equations for the observational and ITT analyses are presented as Eqs. (1) and (2).

1 $$h\left(t\right)\;=\;h_{\mathit0,m}\mathit(t\mathit)\;\times\;\exp\left(\beta_{\mathit1}S^p\;+\;\beta_{\mathit2}B^p\;+\;\beta_{\mathit3}B^o\;+\;\beta_{\mathit4}Sex^p\right)$$

2 $$h\left(t\right)\;=\;h_{\mathit0,m}\left(t\right)\;\times\;\exp\left(\beta_{\mathit1}Z^p\;+\;\beta_{\mathit2}B^p\;+\;\beta_{\mathit3}B^o\;+\;\beta_{\mathit4}Sex^p\right)$$

To estimate the effect of the parental education on offspring outcomes, we implemented a Regression Discontinuity (RD) design [27]. Because years of schooling do not deterministically depend on exposure to the reform (e.g., some individuals acquired 9 or more years of compulsory schooling even before the reform), we applied a fuzzy RD design [28]. The fuzzy RD design corresponds to using the reform as an instrument for parental years of schooling. We employed a continuity-based framework with a two-stage least squares (2SLS) method. This involved regressing parental years of schooling on the reform status (i.e., the first stage; Eq. 3a), and then regressing the outcome variable on the fitted values from the first stage (i.e., the predicted additional years of schooling due to the reform; Eq. 3b):

3a $$S^o\;=\;\alpha_{\mathit0}\;+\;\alpha_{\mathit1}Z\;+\;\alpha_{\mathit2}f\mathit(r\mathit)\;+\;\alpha_{\mathit3}\mathrm Z\ast f\mathit(r\mathit)\;+\;\varepsilon$$

3b $$O^o\;=\;\beta_{\mathit0}\;+\;\beta_{\mathit1}S^p\;+\;\beta_{\mathit2}g\mathit(r\mathit)\;+\;\beta_{\mathit3}Z\ast f\mathit(r\mathit)\;+\;v$$

$$r\;\in\;\left(-h,h\right)$$

In Eqs. 1–3, the superscript *o* denotes the offspring, and *p* denotes the parent. The running variable (*r*) is defined as the difference between parental birth month and the reform cohort year (measured in years, centered at a cutoff of 0) in each municipality. The *f* and *g* are flexible functions (usually an n-order polynomial on each side of the cutoff) of the running variable used to account for trends in education and outcomes. *S*^*p*^ denotes parental years of schooling, *B*^*p*^ denotes parental birth year, *B*^*o*^ denotes offspring birth year, *Sex*^*p*^ denotes parental sex, and *O* represents the offspring outcome. *Z* is a dummy variable indicating whether the parent was exposed to the reform or not (*Z* = 1{*r* ≥ 0}). *h* is a neighborhood around 0, hereby referred to as the bandwidth. The key parameter *β*_*1*_ in Eq. 3b captures the effect of parental education on offspring outcomes for those parents who would have completed only 7 years of education in the absence of the reform (i.e., reform compliers). We corrected the *p*-values for multiple testing (*N* = 17) with the Benjamini–Hochberg false discovery rate for all the above analyses [29].

We employed a local linear regression for the RD effect estimator with one common mean-squared-error-optimal (MSE-optimal) bandwidth selector and robust bias-corrected inference. To ensure the comparability of unexposed and exposed cohorts, observations were weighted by their proximity to the discontinuity using a triangular kernel.

The key identifying assumption of the RD design is that the average potential outcomes are continuous functions of the running variable at the threshold, that is, the pre- and post-reform cohorts (i.e., unexposed and exposed groups) have similar observable and unobservable characteristics except for their exposure status. We tested the continuity of predetermined covariates (parental sex and parental birth year) and the density of the running variable at the reform threshold and found no evidence of significant discontinuities or manipulation around the reform threshold. These findings are consistent with previous research that also found no evidence of selective mobility related to reform [30].

Beyond this standard RD assumption, identification of causal effect requires the same assumptions as IV design. First, the reform exposure must be a sufficiently strong predictor of parental years of schooling. This assumption was clearly met, as indicated by the significant first-stage estimates and large F-statistics (all > 10), reported in the note to Table 2. Second, the reform should affect offspring outcomes exclusively through its effect on parental years of schooling. As this assumption cannot be tested directly, we estimated ITT effects, which capture the total impact of reform without relying on the exclusion restriction. Previous research has documented temporary declines in educational quality immediately after the reform—driven by teacher shortages and organizational disruptions [22, 31]—which could have attenuated the potential benefits of extended schooling and partially violated the exclusion restriction. We tried to address this through sensitivity analysis (see details in Methods section). Third, no individuals reduced their years of schooling due to the reform. While it is theoretically possible that some individuals would otherwise have continued to higher education instead stopped at 9 years when the compulsory minimum was raised, this appears unlikely. In our data, the proportion of individuals completing more than 9 years of schooling for the exposed group was much higher than that for unexposed group (82% vs 74%, see Table 1), suggesting that the monotonicity assumption holds.

**Table 1:** Description of characteristics for unexposed and exposed parents (*N* = 701,350) and their offspring (*N* = 1,047,275)

**Parent-specific characteristics** ***N*(%)**	**Unexposed**	**Exposed**
*N*	350,005	351,345
Mother	171,898	178,785
Father	178,107	172,560
Birth year (mean [SD])	1948 (2.17)	1952 (2.35)
Highest educational level^a^		
Compulsory education of less than 9 years	67,540 (19.30)	7,700 (2.19)
Compulsory education of 9 years	24,323 (6.95)	56,729 (16.15)
Short high school	115,667 (33.05)	125,419 (35.70)
Long high school	45,269 (12.93)	44,376 (12.63)
Short university	41,100 (11.74)	52,160 (14.85)
Long university	52,458 (14.99)	60,557 (17.24)
Postgraduate	3,648 (1.04)	4,404 (1.25)
Years of schooling^b^ (mean [SD])	11.3 (2.85)	12.0 (2.38)
**Offspring-specific characteristics N(%)**		
*N*	479,444	567,831
Sex		
Male	245,126 (51.13)	290,573 (51.17)
Female	234,318 (48.87)	277,258 (48.83)
Birth year		
1970–1980	344,562 (71.87)	250,321 (44.08)
1980–1990	118,596 (24.74)	259,352 (45.67)
1990–2000	16,286 (3.40)	58,158 (10.24)
Any psychiatric diagnosis	68,405 (14.27)	89,173 (15.70)
Bipolar disorder	4272 (0.89)	5296 (0.93)
Schizophrenia	1504 (0.31)	1549 (0.27)
ADHD	5862 (1.22)	10,082 (1.78)
Tic disorder	474 (0.10)	777 (0.14)
ASD	2710 (0.57)	4529 (0.80)
Intellectual disability	2557 (0.53)	3342 (0.59)
Learning disorders	498 (0.10)	1025 (0.18)
Anxiety	23,786 (4.96)	30,959 (5.45)
Depression	24,707 (5.15)	31,141 (5.48)
PTSD	14,906 (3.11)	16,793 (2.96)
OCD	3238 (0.68)	4215 (0.74)
Alcohol-related disorders	7180 (1.50)	8972 (1.58)
Drug-related disorders	7585 (1.58)	11,022 (1.94)
ODD	446 (0.09)	827 (0.15)
Violent crimes	14,716 (3.07)	20,599 (3.63)
Suicidal behavior	10,097 (2.11)	15,038 (2.65)

*ADHD* attention deficit hyperactivity disorder, *ASD* autism spectrum disorder, *PTSD* posttraumatic stress disorder, *OCD* obsessive–compulsive disorder, *ODD* oppositional defiant disorder

^a^Short high school corresponds to upper secondary education of less than 3 years, long high school corresponds to upper secondary education of 3 years, short university corresponds to post-secondary education of less than 3 years, long university corresponds to post-secondary education of 3 years or more

^b^Years of schooling represented the average number of years corresponding to the educational attainment level

### Sensitivity analyses

We conducted six sensitivity analyses.

To assess the robustness of the results, we first conducted analyses under alternative specifications of the continuity-based framework. We systematically varied (1) bandwidth selection (MSE-optimal vs. coverage-error-rate-optimal), (2) kernel weighting functions (triangular vs. uniform), and (3) local polynomial orders (linear vs. quadratic). To complement the continuity-based approach, we also employed the local randomization framework, which relies on the assumption of local random assignment of the treatment near the cutoff. We used parental sex as a predetermined covariate.

Second, to examine whether the results remained consistent when examining the full range of observations around the cut-off, we employed a parametric 2SLS IV framework, that is, a global linear model. We adjusted for parental birth year, sex, municipality, and offspring birth year by adding dummy variables. We estimated robust SEs by clustering on the family level.

Third, while we could not identify compliers who would have completed only 7 years of education in the absence of the reform, we restricted the analyses to individuals who completed only compulsory schooling, as compliers were less likely to have pursued higher education only because of the reform. We note that although this approach does not capture all reform compliers (as some might have pursued education beyond the compulsory level), it likely contains a larger proportion of compliers. We hypothesized that, if any effect were observed, it would be more pronounced in this subsample.

Fourth, because the education reform was implemented over such a long period of time, this might raise concerns about the comparability of the exposed and unexposed groups. Although we adjusted for birth year in the main analyses, the assumption that individuals start school exactly at age 7 may not always be true, such that we might have misclassified individuals born around the reform cohort year. Therefore, we restricted the analysis to individuals born at most five years before or after the reform cohort year to make exposed and unexposed groups more comparable, and excluded those born on or one year before the reform cohort year to reduce potential misclassification.

Fifth, because we treated the time-to-event outcomes as continuous variables in the IV analysis, we stratified our analysis by offspring birth year (1970–1980, 1980–1990, 1990–2000) to ensure that offspring had roughly the same follow-up time.

Sixth, to examine sex differences, we analyzed the associations separately by fathers and mothers, female offspring, male offspring, and for the specific parent–offspring pairs: father-son, father-daughter, mother-son, and mother-daughter.

Seventh, to examine whether the effect differed depending on whether both parents were exposed, we conducted a sensitivity analysis restricted to families in which both parents were exposed versus both unexposed to the reform.

Data management was performed using SAS version 9.4. Data analyses were performed using R version 4.4.3 with the packages “survival,” “fixest,” “rdrobust,” and “rdlocrand.” RD plots were generated by using the package “rdplot.”

## Results

The final sample consisted of 701,350 parents from 926 municipalities and their 1,047,275 offspring, comprising 1,459,643 parent–child pairs. Among the parents, 350,005 were unexposed to the reform, and 351,345 were exposed. Table 1 presents the distribution of characteristics of exposed and unexposed parents and their offspring. We observed that the reform not only increased the proportion of parents completing 9 years of compulsory education from 6.95% to 16.15%, but also slightly increased the proportion of parents completing higher education, suggesting that the reform may have had a small spillover effect beyond the minimum education requirement.

### Observational association between parental years of schooling and offspring outcomes

Table 2 (column 2) shows that longer years of parental schooling were significantly associated with a lower risk for any psychiatric diagnosis in their offspring, with a HR of 0.97 (95% CI 0.97, 0.97). This negative association was observed for most of the 14 psychiatric diagnoses, violent crime, and suicidal behavior, with HRs ranging from 0.87 to 0.99. However, parental years of schooling were associated with a slightly increased risk of bipolar disorder (HR = 1.01; 95% CI 1.01, 1.02) and schizophrenia (HR = 1.04; 95% CI 1.02, 1.05) in the offspring.

**Table 2:** The observational estimates, intent-to-treat estimates, and regression discontinuity estimates of parental years of schooling on offspring outcomes

Offspring outcome	Observational estimates HR (95% CI)	Intent-to-treat estimates HR (95% CI)	Regression discontinuity estimates_a_ *β* (95% CI)
Any psychiatric diagnosis	0.97 (0.97, 0.97)	1.00 (0.99, 1.02)	0.001 (− 0.018, 0.017)
Bipolar disorder	1.01 (1.01, 1.02)	1.00 (0.94, 1.07)	− 0.001 (− 0.005, 0.004)
Schizophrenia	1.04 (1.02, 1.05)	1.03 (0.92, 1.15)	− 0.001 (− 0.004, 0.002)
ADHD	0.93 (0.92, 0.93)	0.98 (0.93, 1.03)	− 0.001 (− 0.007, 0.005)
Tic disorder	0.97 (0.95, 0.99)	1.07 (0.89, 1.29)	0.000 (− 0.002, 0.001)
ASD	0.99 (0.98, 1.00)	1.01 (0.94, 1.09)	0.000 (− 0.005, 0.003)
Intellectual disability	0.87 (0.86, 0.88)	0.90 (0.83, 0.98)	0.000 (− 0.003, 0.004)
Learning disorders	0.90 (0.88, 0.92)	1.14 (0.96, 1.34)	0.001 (− 0.001, 0.003)
Anxiety	0.97 (0.97, 0.97)	1.00 (0.97, 1.02)	0.009 (− 0.002, 0.020)
Depression	0.99 (0.98, 0.99)	1.01 (0.99, 1.04)	0.004 (− 0.009, 0.014)
PTSD	0.95 (0.95, 0.96)	0.99 (0.95, 1.02)	0.004 (− 0.004, 0.013)
OCD	1.01 (1.00, 1.02)	1.00 (0.93, 1.07)	− 0.001 (− 0.006, 0.002)
Alcohol-related disorders	0.94 (0.93, 0.94)	1.02 (0.97, 1.07)	0.001 (− 0.006, 0.007)
Drug-related disorders	0.92 (0.91, 0.92)	1.03 (0.98, 1.08)	0.002 (− 0.005, 0.009)
ODD	0.87 (0.85, 0.89)	1.05 (0.87, 1.26)	0.000 (− 0.001, 0.002)
Violent crimes	0.88 (0.87, 0.88)	1.00 (0.96, 1.03)	− 0.012 (− 0.021, − 0.004)
Suicidal behavior	0.94 (0.94, 0.94)	0.98 (0.95, 1.01)	− 0.005 (− 0.014, 0.004)

Observational estimates refer to the association between parental education and offspring outcomes estimated using a stratified Cox regression model, adjusting for parental birth year, sex, and offspring birth year, with municipality as a stratification variable and robust standard errors clustered at the family level (corresponding Eq. 1). Intent-to-treat estimates correspond to the reduced-form effects of reform on offspring outcomes, estimated using the same stratified Cox regression model as observational estimates (corresponding Eq. 2). Regression discontinuity estimates capture the causal effect of parental education on offspring outcomes by using reform as an instrument for parental education (corresponding Eq. 3). The sample size for the observational analyses, intent-to-treat analyses, and regression discontinuity analyses was 1,459,643 parent–child pairs. However, the effective sample size contributing to each regression discontinuity estimate varies by outcome because it is determined by the outcome-specific bandwidth (selected using a common mean-squared-error-optimal bandwidth selector). The corresponding bandwidths and effective sample sizes are reported in Table S3 in the Additional file 1

*ADHD* attention deficit hyperactivity disorder, *ASD* autism spectrum disorder, *PTSD* posttraumatic stress disorder, *OCD* obsessive–compulsive disorder, *ODD* oppositional defiant disorder

^a^All first-stage F-statistics are well above the conventional threshold of 10 (mean 244, range 230–261), indicating strong instrument relevance

### Intent-to-treat estimates of education reform on offspring outcomes

**Fig. 1:**
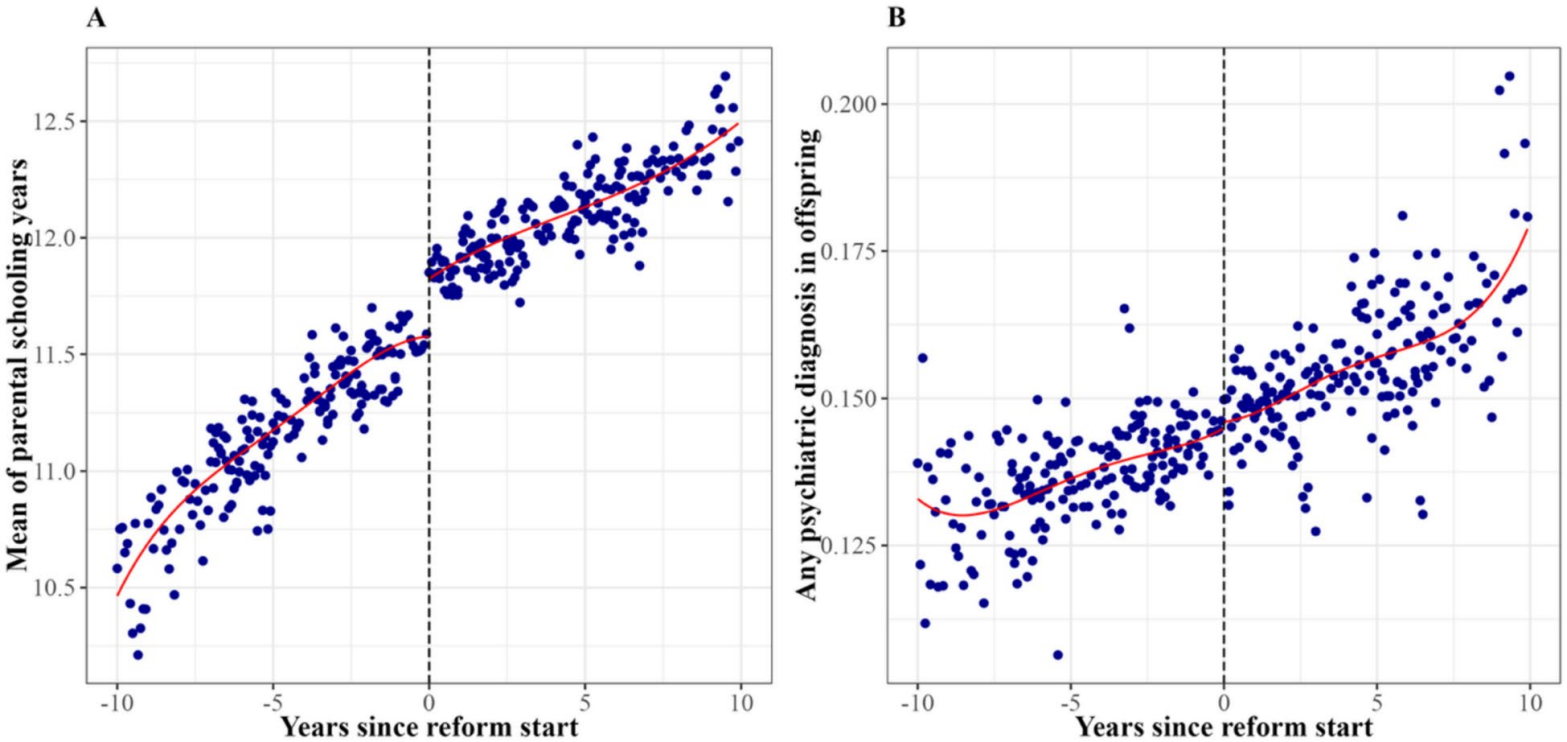
Parental education and offspring psychiatric diagnosis by years since reform implementation. Note: Points represent local averages of parental years of schooling (**A**) and offspring psychiatric diagnosis (**B**), aggregated by parental birth month relative to the reform cohort year. Curves represent fourth-order polynomial fits. The *x*-axis is centered at 0, corresponding to parents born in the reform cohort year. The sample size used for plotting **A** was 701,350, and the sample size used for plotting **B** was 1,459,643. The estimated first stage effect (**A**) of the reform on parental years of schooling was 0.230 [95% CI 0.211, 0.272], indicating a statistically significant increase in years of schooling

Figure 1a shows a clear discontinuity in parental years of schooling at the reform threshold, with the average years of schooling increasing from 11.3 years for those born before the reform to 12 years for those born after (Table 1). However, as shown in Fig. 1b, we observed no apparent difference in the probability of any psychiatric diagnosis among the offspring of parents unexposed and exposed to the reform (no apparent differences were observed for all the other outcomes; see Figure S1 in Additional file 1). Consistently, Table 2 (column 3) shows ITT estimates between the educational reform and the offspring outcomes, which were generally clustered near the null value (HR = 1.0) and did not reach statistical significance. The only exception was a significant 10% reduction in the risk of intellectual disability (HR = 0.90; 95% CI 0.83–0.98), but this association was not statistically significant after multiple testing corrections.

### Regression discontinuity estimates of parental years of schooling on offspring outcomes

Table 2 (column 4) shows the fuzzy RD estimates of parental years of schooling on the offspring outcomes. In contrast to the negative observational associations, when instrumented by the education reform, parental years of schooling were no longer significantly associated with offspring psychiatric diagnoses or suicidal behavior. The only exception was observed for violent crimes (*β* = − 0.012, 95% CI − 0.021, − 0.004); however, this association was not statistically significant after correction for multiple testing.

### Sensitivity analyses

All seven sensitivity analyses generated results consistent with the main analysis (Figure S2 in Additional file 1). Although some associations remained or emerged (e.g., negative associations with violent crimes were observed under alternative fuzzy RD specifications, and in maternal, male offspring, and mother-son subsample; negative associations with schizophrenia and suicidal behavior were observed among compliers; positive associations with anxiety were observed in maternal, male offspring, and mother-son subsample; positive association with alcohol-related disorders was observed in father-daughter subsample; and positive association with anxiety in doubly exposed-parents subsample), none of these remained statistically significant after correcting for multiple testing.

## Discussion

Children of more educated parents exhibited a lower risk of psychiatric diagnoses, violent crimes, and suicidal behavior. However, children with parents exposed to the Swedish education reform, which extended compulsory schooling from 7 to 9 years, displayed no significant reductions in these outcomes, compared to children with parents who were not exposed to the reform. RD analyses (leveraging the reform as an instrument) confirmed the null effects, replicating two previous reform studies that relied on self-reported mental health service utilization or mental conditions [7, 19], and extending them to 14 clinically recorded psychiatric diagnoses, violent crimes, and suicidal behavior in adulthood. Unlike the significant mother-daughter effect in the German education reform study [21], our sex-stratified analyses did not reveal any sex differences.

Previous studies using IVs and family-based designs reported null effects of parental education on a global self-reported mental health index, depression, and anxiety in the offspring, suggesting that the null effect replicates across both surveys and electronic health records [12, 13, 16, 17, 32]. Notably, studies of compulsory schooling reforms in the UK, USA, and Denmark also observed null effects on other developmental and health outcomes in the offspring, including cognitive development [33, 34], non-cognitive development [34], health care utilization [7], and long-term illness [35]. While we find no quasi-causal effect of parental education, some quasi-experimental studies of other socioeconomic factors—such as childhood family income and neighborhood deprivation—have reported mixed effects on offspring psychiatric disorders, substance misuse, and violent crime [36–41]. Thus, in future quasi-experimental studies, it might be beneficial to further focus on such SES-related constructs (rather than parental education per se).

However, as the reform only increased compulsory schooling, the null effects might be attributable to a lack of increase in secondary or tertiary education. Past research indicates that a higher education level is relevant for improving parental socioeconomic status, parenting practices, health outcomes, or offspring educational attainment, all of which could mediate mental health problems [19, 31, 42, 43]. In addition, in Sweden, the earnings distribution is relatively compressed due to generous welfare services; the mental health consequences of social disadvantage may be more pronounced in societies with greater structural inequalities. For example, a study using the Universal Primary Education Reform in Uganda as an instrument for maternal schooling found positive effects on parenting practices and children’s early educational engagement, which partially mediated early childhood cognitive and emotional development [44]. Furthermore, the null effect could also reflect opposing forces that cancel out. For instance, increased parental education may both improve health literacy—leading to greater awareness and help-seeking and consequently higher detection and diagnosis rates—and enhance socioeconomic resources, which could reduce family stress and the risk of mental health problems.

Our findings appear to contrast with one children-of-twins/siblings study, which reported a significant effect of parental educational attainment on offspring ADHD, after accounting for shared genetic and environmental factors [13]. Our null effects on behavioral problems also differ from a previous study using local educational infrastructure variables as instruments, which found that maternal education reduced parent-reported childhood behavioral problems [18]. In addition, unlike the significant effect found between maternal education and daughters’ mental health in a German study, our sex-stratified analyses did not reveal such a pattern [21]. These discrepancies may stem from differences in outcome measurement (i.e., clinically recorded diagnoses vs. parent/self-reported symptoms). Alternatively, they might stem from differences in quasi-experimental methodologies: infrastructure as an instrument might capture broader socioeconomic dynamics (e.g., community resources, economic opportunities) than education reform, and family studies might be subject to residual genetic and non-shared unmeasured confounding. Furthermore, our estimates represent local average treatment effects specific to marginal increases in parental education at the lower end of the distribution—that is, among individuals who would otherwise have completed only 7 years of schooling in the absence of the reform. Differences in the populations and educational margins targeted by various quasi-experimental designs may therefore contribute to the observed discrepancies.

## Limitations

Although our study assessed the effect of parental education on a wide range of registry-based psychiatric, criminal, and suicidal outcomes using a nationwide education reform, the results should be interpreted in light of several limitations. First, as noted above, our results are specific to marginal increases in parental education at the lower end of the educational distribution, representing the local average treatment effect for individuals who would otherwise have completed only 7 years of schooling in the absence of reform. These results do not generalize to marginal increases in higher educational attainment (e.g., post-compulsory or tertiary education), which may yield stronger labor market returns and enhance child health productivity. On a related note, as our estimates capture the average treatment effect among compliers, this may mask meaningful differences across subgroups. It is possible that the reform benefited some offspring while adversely affecting others, resulting in an overall null average effect. Second, the Swedish compulsory schooling reform was implemented in the mid-twentieth century, and secular trends in education systems, labor markets, and mental health awareness may limit the temporal generalizability of our findings to contemporary populations. Third, although registry-based outcomes reduce reporting bias, they likely capture more severe cases treated in specialist care or that adjudicated by the court, and fewer individuals with milder mental health symptoms that are commonly treated in primary care, subclinical conditions, behavioral issues that do not result in legal consequences or problems for which individuals do not seek help. Fourth, there is no statistical package for conducting fuzzy RDs for binary and time-to-event outcomes, so we treated outcomes as continuous variables and used a local linear probability model (as has been done in previous studies [7]). Although the linear probability model may perform poorly for rare outcomes, we also estimated the ITT effects by treating the outcomes as time-to-event variables and the null effects observed were consistent with the RD analysis. Fifth, the observed null effects may stem from a countervailing mechanism: post-reform declines in educational quality (evidenced by immediate teacher shortages and institutional disorganization following implementation) could have offset the potential benefits of prolonged compulsory schooling [22, 31]. Nevertheless, sensitivity analysis excluding cohorts born within 1-year post-reform suggests that these pathways are unlikely to fully account for the null results. Sixth, the observed null effect of the reform could be attenuated if one parent was affected by the reform while the other was not. However, our analysis restricting to families in which both parents were exposed versus both unexposed to the reform showed consistent null effects across all offspring mental health problems and behavioral outcomes.

## Conclusions

Although parental years of education were significantly associated with a reduced risk of offspring mental health problems and behavioral outcomes, this seemed attributable to unmeasured confounding.

## Supplementary Information

Additional File 1: Tables S1-S2, Figures S1-S2. Table S1: Description of registries and variables extracted. Table S2: The ICD code, classified convictions for violent crimes, and the cut-off age for each outcome. Table S3: Bandwidth and effective sample size for main regression discontinuity analysis. Figure S1: Parental education and offspring psychiatric diagnosis, violent crimes, and suicidal behavior by years since reform implementation. Figure S2: Quasi-causal estimates of parental years of schooling on offspring outcomes, sensitivity analyses [45–50].

## Data Availability

The Public Access to Information and Secrecy Act in Sweden prohibits us from making individual-level data publicly available. Researchers can apply for individual-level data through Statistics Sweden at https://www.scb.se/en/services/guidancefor-researchers-and-universities/.

## Abbreviations

ADHD: Attention deficit hyperactivity disorder
ASD: Autism spectrum disorder
OCD: Obsessive-compulsive disorder
PTSD: Posttraumatic stress disorder
ODD: Oppositional defiant disorder
IV: Instrumental variable
HR: Hazard ratio
SE: Standard errors
ITT: Intent-to-treat
2SLS: Two-stage least squares
MSE-optimal: Mean-squared-error-optimal
