# Supplementary material for: Association of parental education with offspring psychiatric diagnoses, violent crimes, and suicidal behavior: a nationwide Swedish quasi-experimental study
Source: BMC Med. 2026 Feb 26;24:133. doi: 10.1186/s12916-026-04719-w (PMC12955071; doi:10.1186/s12916-026-04719-w)
Supplement: Supplementary file 1 — Additional File 1: Tables S1-S2, Figures S1-S2. Table S1: Description of registries and variables extracted. Table S2: The ICD code, classified convictions for violent crimes, and the cut-off age for each outcome. Table S3: Bandwidth and effective sample size for main regression discontinuity analysis. Figure S1: Parental education and offspring psychiatric diagnosis, violent crimes, and suicidal behavior by years since reform implementation. Figure S2: Quasi-causal estimates of parental years of schooling on offspring outcomes, sensitivity analyses [45–50]. [file 12916_2026_4719_MOESM1_ESM.docx]

**Article title**

Association of parental education with offspring psychiatric diagnoses, violent crimes, and suicidal behavior: A nationwide Swedish quasi-experimental study

**Authors**

Mengping Zhou MSc^1^, Henrik Larsson PhD^1,2^, Brian M. D’Onofrio PhD^1,3^, Mikael Landén PhD^1,4^, Paul Lichtenstein PhD^1^, Erik Pettersson PhD^1^

^1^ Department of Medical Epidemiology and Biostatistics, Karolinska Institutet, Stockholm, Sweden

^2^ School of Medical Sciences, Örebro University, Örebro, Sweden

^3^ Department of Psychological and Brain Sciences, Indiana University, Bloomington, IN, USA

^4^ Institute of Neuroscience and Physiology, The Sahlgrenska Academy at Gothenburg University, Gothenburg, Sweden

**Supplementary Content**

**Table S1.** Description of registries and variables extracted

**Table S2.** The ICD code, classified convictions for violent crimes, and the cut-off age for each outcome

**Table S3.** Bandwidth and effective sample size for main regression discontinuity analysis

**Figure S1.** Parental education and offspring psychiatric diagnosis, violent crimes, and suicidal behavior by years since reform implementation

**Figure S2.** Quasi-causal estimates of parental years of schooling on offspring outcomes, sensitivity analyses

**Table S1. Description of registries and variables extracted**

| **Register** | **Description** | **Variables** |
| --- | --- | --- |
| Total Population Register | Established in 1968 and includes demographic information (e.g., sex, age, place of birth) for the entire Swedish population. [45] | Individual identification number, birthyear, sex |
| Multi-Generation Register | Links all index persons born in Sweden since 1932 and alive in 1960 to their biological or adoptive parent. [46] | Individual identification number |
| National Patient Register | Includes individual-based records of psychiatric inpatient care since 1973 (complete since 1987) and psychiatric outpatient care since 2001 (complete since 2010). All diagnoses were recorded according to the International Classification of Diseases (ICD); 8th (1973/1969-1986), 9th (1987-1996), and 10th (1997-2009) revision. [47,48] | Disorders, suicide attempts |
| National Crime Register | Comprises all registered criminal convictions or suspicion of those aged 15 and older (the age of criminal responsibility) since 1973. [49] | Court convictions of violent crimes |
| Cause of Death Register | Records all deaths in Sweden since 1952 and provides information on causes of death according to ICD. [50] | Death by suicide |
| Population and Housing Census | Information about employment, households, and accommodation, has been carried out every fifth year since 1960. | Municipality, educational attainment |

**Table S2. The ICD code, classified convictions for violent crimes, and the cut-off age for each outcome**

| **Exposure/outcome** | **ICD 08 (1969-1986)** | **ICD 09 (1987-1996)** | **ICD 10 (1997-)** | **Minimum age** |
| --- | --- | --- | --- | --- |
| Bipolar disorder | 296.1, 296.3, 296.8, 296.9 | 296A, 296C, 296D, 296E, 296W, 296X | F30, F31 | 15 |
| Schizophrenia | 295 (except 295.5) | 295 (except 295F) | F20, F25 | 15 |
| ADHD | - | 314 | F90 | 3 |
| Tic disorder | 306.2 | 307C | F95 | 3 |
| ASD | - | 299A | F84.0, F84.1, F84.5 | 2 |
| Intellectual disability | 311-315 | 317-319 | F70-F79 | 2 |
| Learning disorders | - | 315A, 315B | F81 | 3 |
| Anxiety | 300 (except 300.3, 300.4) | 300, (except 300E, 300D) | F40, F41 | 10 |
| Depression | 300.4 | 296B, 311, 300E | F32, F33 | 10 |
| PTSD | 307.99 | 308, 309 | F43 | 2 |
| OCD | 300.3 | 300D | F42 | 5 |
| Alcohol-related disorders | 291, 303 | 291, 303, 305A | F10 (except F10.5) | 12 |
| Drug-related disorders | 304 | 292, 304, 305X | F11-F19 (except F17, F1x.5) | 12 |
| ODD | - | 312X | F91 | 3 |
| Suicidal behavior (Suicide attempts and death by suicide) | E950-959 E980-989 | E950-959 E980-989 | X60-X84, Y10-Y34 | 10 |
| Violent crimes | homicide (Ch 3, §1-3); assault (Ch 3, §5-6); robbery (Ch 8, §5-6); threats and violence against an officer (Ch 17, §1-2); gross violation of a person’s/woman’s integrity (Ch 4, §4a); unlawful coercion (Ch 4, §4); unlawful threats (Ch 4, §5); kidnapping (Ch 4, §1); illegal confinement (Ch 4, §2); arson (Ch 13, §1-2); intimidation (Ch 4, §7); sexual offence (excluding prostitution and the buying of sexual services but including child pornography) (Ch 6 §1-10, §10A, §12) | | | 15 |

**Table S3. Bandwidth and effective sample size for main regression discontinuity analysis**

| **Offspring outcome** | **Bandwidth** | **Sample size** |
| --- | --- | --- |
| Any psychiatric diagnosis | 2.2 | 570,484 |
| Bipolar disorder | 3.1 | 764,614 |
| Schizophrenia | 2.5 | 649,893 |
| ADHD | 2.6 | 669,372 |
| Tic disorder | 2.0 | 533,265 |
| ASD | 2.3 | 590,596 |
| Intellectual disability | 2.8 | 710,845 |
| Learning disorders | 2.7 | 689,188 |
| Anxiety | 2.3 | 590,596 |
| Depression | 2.4 | 630,810 |
| PTSD | 3.0 | 746,765 |
| OCD | 1.9 | 493,562 |
| Alcohol-related disorders | 2.3 | 590,596 |
| Drug-related disorders | 2.5 | 649,893 |
| ODD | 2.8 | 709,165 |
| Violent crimes | 2.2 | 570,484 |
| Suicidal behavior | 2.2 | 570,484 |


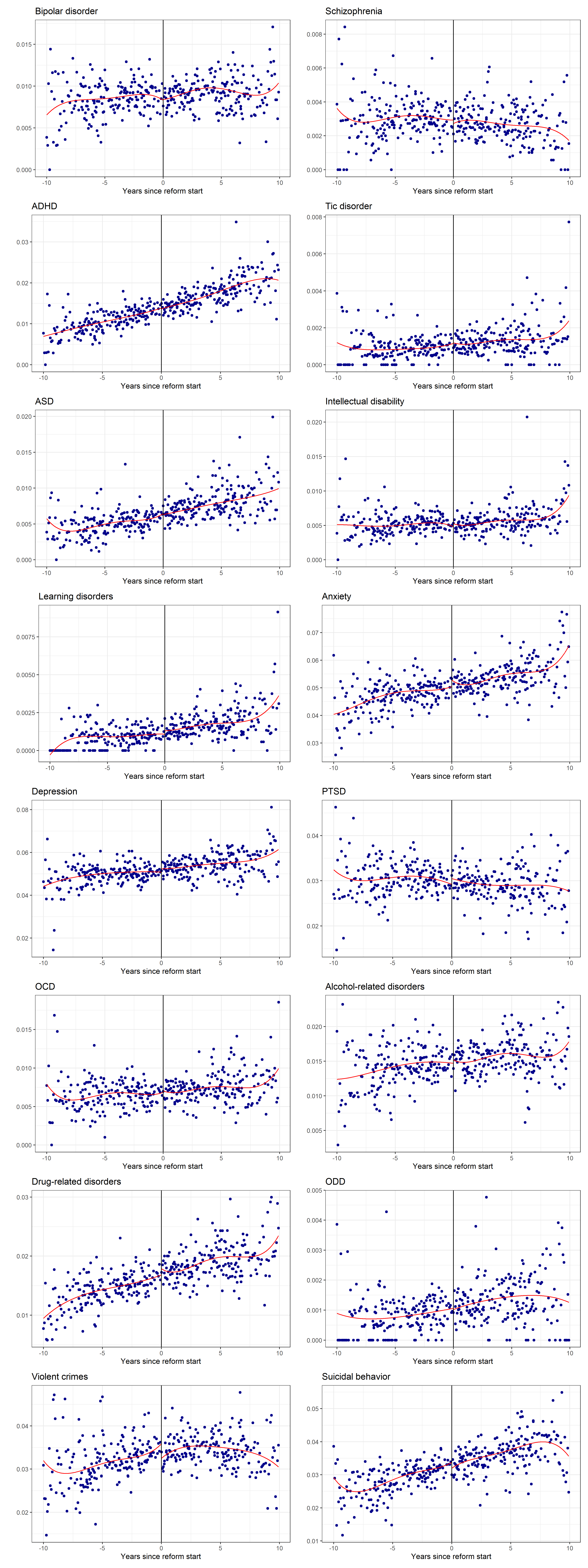


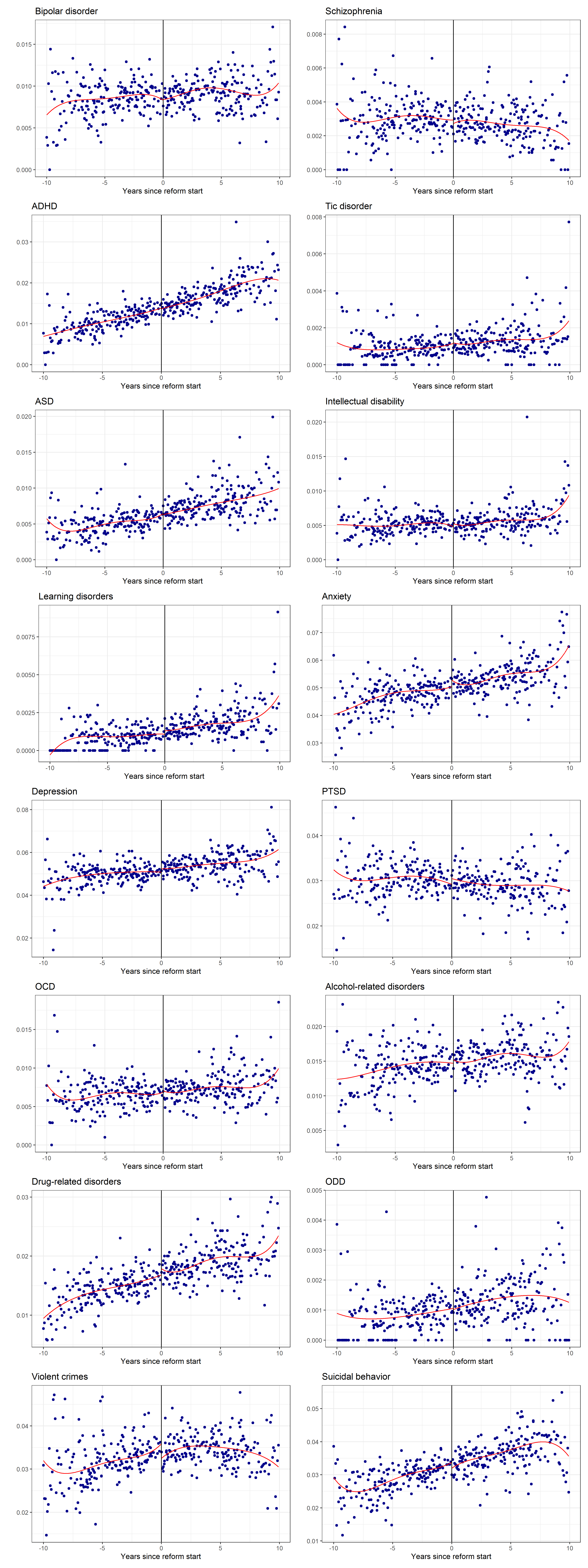


**Figure S1. Parental education and offspring psychiatric diagnosis, violent crimes, and suicidal behavior by years since reform implementation**


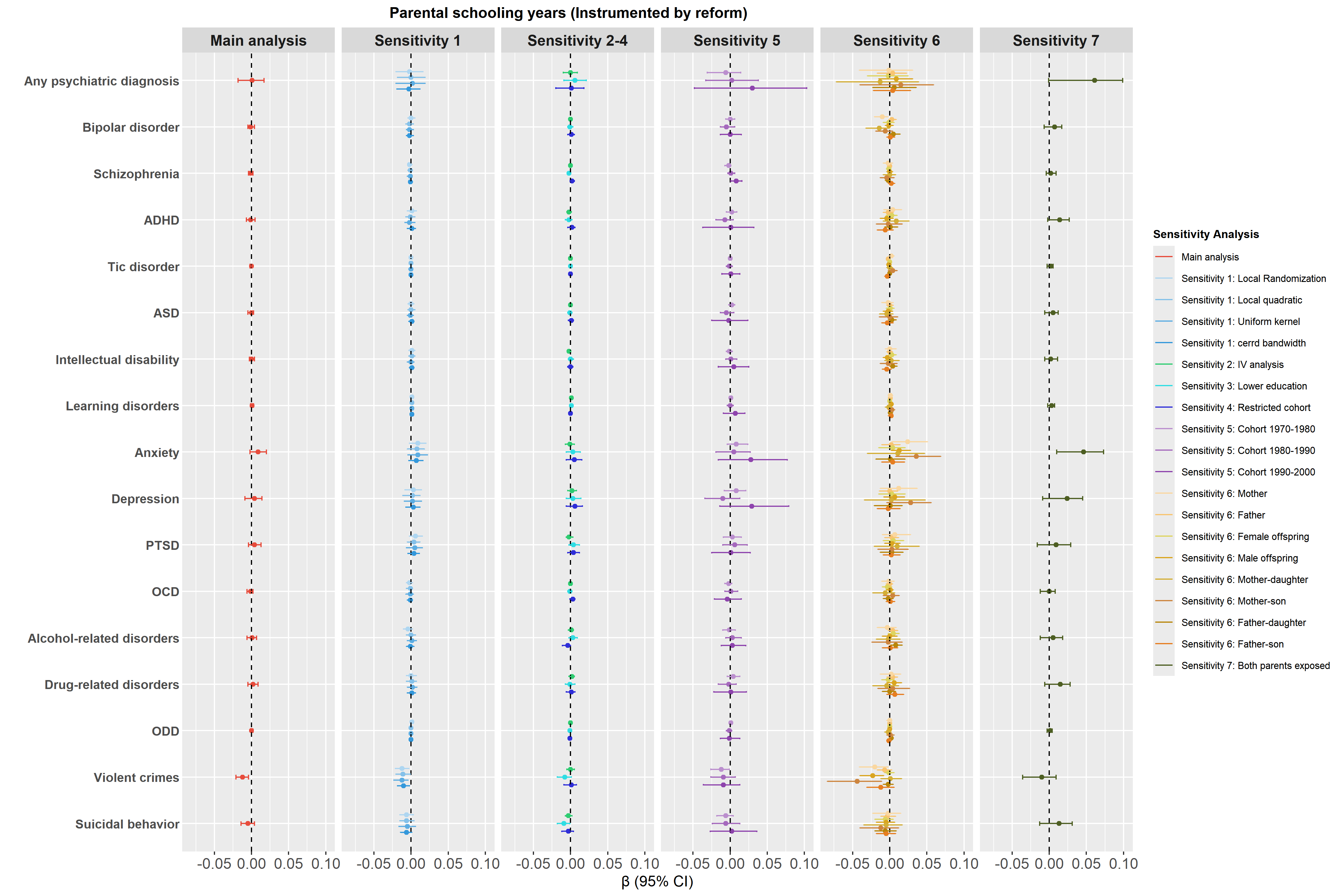


**Figure S2. Quasi-causal estimates of parental schooling years on offspring outcomes, sensitivity analyses**

Note: The model setting for the main analysis is one common mean-squared-error-optimal bandwidth (bwselect = mserd), triangular kernel function (kernel = triangular), local linear regression to construct the point-estimator (p = 1), and local quadratic regression to construct the bias-correction (q = 2). cerrd = Coverage Error Rate-optimal bandwidth selector, ADHD = attention deficit hyperactivity disorder, ASD = autism spectrum disorder, PTSD = posttraumatic stress disorder, OCD = obsessive-compulsive disorder, ODD = oppositional defiant disorder. The sample size for the main regression discontinuity analysis and Sensitivity Analyses 1 and 2 was 1,459,643 parent-child pairs. The analytic samples for the remaining sensitivity analyses were as follows: Sensitivity Analysis 3: 317,019; Sensitivity Analysis 4: 857,408; Sensitivity Analysis 5 - Cohort 1970-1980: 789,364; Cohort 1980-1990: 558,089; Cohort 1990-2000: 111,989; Sensitivity Analysis 6 - mother: 706,698; father: 752,744; female offspring: 713,077; male offspring: 746,566; mother-daughter: 345,595; mother-son: 361,178; father-daughter: 367,482; father-son: 385,388. 7: 541,064 parent-child pairs.
